# Pericardial Effusion on MRI in Autosomal Dominant Polycystic Kidney Disease

**DOI:** 10.3390/jcm11041127

**Published:** 2022-02-21

**Authors:** Jin Liu, Kana Fujikura, Hreedi Dev, Sadjad Riyahi, Jon Blumenfeld, Jiwon Kim, Hanna Rennert, Martin R. Prince

**Affiliations:** 1 Department of Radiology, Weill Cornell Medicine, New York, NY 10065, USA; jil4018@med.cornell.edu (J.L.); hrd2001@med.cornell.edu (H.D.); sar4002@med.cornell.edu (S.R.); 2 Department of Geriatrics, The Second Affiliated Hospital of Nanjing Medical University, Nanjing 210011, China; 3 Department of Cardiology, Saint Francis Hospital, New York, NY 11576, USA; kana.fujikura@chsli.org; 4 Department of Medicine, Weill Cornell Medicine, New York, NY 10065, USA; jdblume@nyp.org (J.B.); jik9027@med.cornell.edu (J.K.); 5 The Rogosin Institute, New York, NY 10065, USA; 6 Department of Pathology, Weill Cornell Medicine, New York, NY 10065, USA; har2006@med.cornell.edu; 7 Columbia College of Physicians and Surgeons, New York, NY 10027, USA

**Keywords:** ADPKD, pericardial effusion, MRI, echocardiography, pleural effusion

## Abstract

Autosomal dominant polycystic kidney disease (ADPKD) has been associated with cardiac abnormalities including mitral valve prolapse and aneurysmal dilatation of the aortic root. Herein, we investigated the potential association of pericardial effusion with ADPKD. Subjects with ADPKD (*n* = 117) and control subjects without ADPKD matched for age, gender and renal function (*n* = 117) undergoing MRI including ECG-gated cine MRI of the aorta and heart were evaluated for pericardial effusion independently by three observers measuring the maximum pericardial effusion thickness in diastole using electronic calipers. Pericardial effusion thickness was larger in ADPKD subjects compared to matched controls (Mann–Whitney *p* = 0.001) with pericardial effusion thickness >5 mm observed in 24 of 117 (21%) ADPKD subjects compared to 4 of 117 (3%) controls (*p* = 0.00006). Pericardial effusion thickness in ADPKD was associated with female gender patients (1.2 mm greater than in males, *p* = 0.03) and pleural effusion thickness (*p* < 0.001) in multivariate analyses. No subjects exhibited symptoms related to pericardial effusion or required pericardiocentesis. In conclusion, pericardial effusion appears to be more prevalent in ADPKD compared to controls. Although in this retrospective cross-sectional study we did not identify clinical significance, future investigations of pericardial effusion in ADPKD subjects may help to more fully understand its role in this disease.

## 1. Introduction

Autosomal dominant polycystic kidney disease (ADPKD) is the most common hereditary cause of chronic kidney disease [1,2,3] and affects the cardiovascular system with hypertension, intracranial (berry) aneurysms, dolichoectasia of the carotid arteries, mitral valve prolapse and aneurysmal dilatation of the aortic root and proximal aorta [4,5,6,7,8]. We have incidentally noted pericardial effusions in ADPKD subjects while evaluating magnetic resonance imaging (MRI) of research subjects in the Rogosin Institute PKD Repository. In this study, we determined the prevalence of pericardial effusion in ADPKD subjects on MRI compared to a control population without ADPKD matched for age, gender and kidney function.

## 2. Materials and Methods

This HIPAA-compliant study was approved by the local Institutional Review Board at Weill Cornell Medicine. All subjects in the Rogosin Institute PKD Repository signed informed consent.

### 2.1. Study Design and Populations

**Figure 1:**
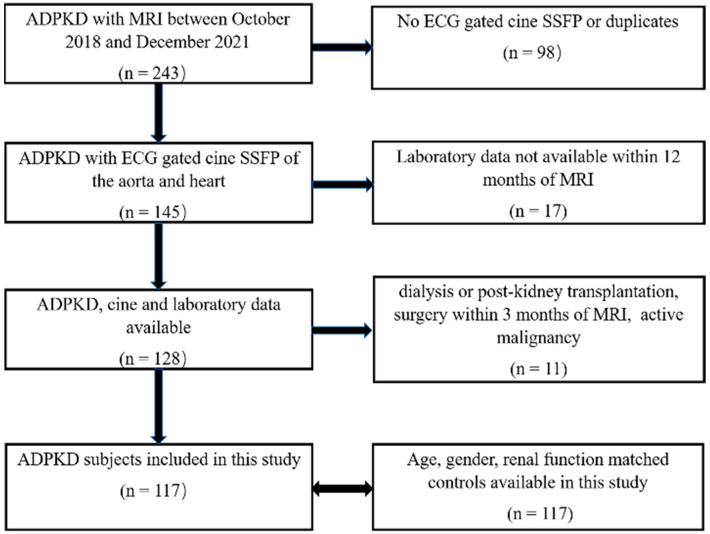
Study flow chart.

This is a retrospective cross-sectional study of existing data and images acquired from October 2018 to December 2021. Inclusion criteria were (1) diagnosis of ADPKD based upon Pei criteria [9,10] and (2) MRI including ECG-gated cine steady-state free precession (SSFP) of the aorta and heart. Exclusion criteria were (1) absence of ECG-gated cardiac images, (2) laboratory data not available within 12 months of the MRI, (3) dialysis or kidney transplantation, (4) surgery within 3 months of MRI and (5) active malignancy (Figure 1).

Age, gender and estimated glomerular filtration rate (eGFR)-matched control subjects without ADPKD undergoing cardiac MRI were identified from electronic medical records, including the picture archiving computer system (PACS). Additionally, subjects were excluded if they had risk factors for pericardial effusion such as pericarditis, ejection fraction less than 50%, active malignancy or surgery within 3 months of MRI, none of which were seen in the ADPKD group. Subjects with hypothyroidism were also excluded from the control population. Race, when available, was also matched.

### 2.2. Data Extraction

Demographic and laboratory data were extracted from electronic medical records for the date closest to the date of MRI. Data on kidney, liver and spleen volumes and cisterna chyli diameter were extracted from the MRI reports prepared prospectively at the time of imaging. The PKD gene mutation was obtained from the Rogosin Institute PKD Repository. Serum creatinine, blood urea nitrogen (BUN), thyroid-stimulating hormone (TSH), alanine transaminase (ALT), aspartate transaminase (AST) and albumin tests were performed within 1 year of the protocol MRI. eGFR was calculated by the CKD-EPI equation [11]. Data on troponin, copeptin and natriuretic peptides were not available. Where available, data on fluid intake were obtained from the electronic medical records, grouping patients into <3 L/day or “increased”, ≥3 L/day of fluid intake. Data on use of diuretics, tolvaptan and other medications, history of rheumatological disease as well as indications for cardiac MRI in control subjects were obtained from the electronic medical record. Data on echocardiography were extracted from tests performed within 1 year of MRI.

### 2.3. Image Acquisition

All MRI exams were obtained on a 1.5 T using a body array coil (Signa HDXT, GE Healthcare, Waukesha, WI, USA, or Magnetom Aera, Siemens Healthineers, Erlangen, Germany) using the parameters shown in Table 1. Pulse sequences included, coronal and axial T2-weighted single-shot fast spin echo (SSFSE), 3D spoiled gradient-recalled echo T1-weighted images with fat suppression or Dixon fat–water separation, diffusion weighted imaging (DWI) and sagittal oblique (in the plane of aortic arch) ECG-gated SSFP of the heart and aorta. The cine images were acquired in a single breath hold per slice, reconstructing the R-to-R interval into 20 temporal phases.

Transthoracic echocardiography was performed using commercial equipment standardized for routine clinical practice. Parasternal long axis, parasternal short axis, apical 4-chamber, apical 2-chamber, apical 3-chamber, subcostal, and suprasternal notch views were acquired in accordance with consensus guidelines [12].

### 2.4. Image Analysis

**Figure 2:**
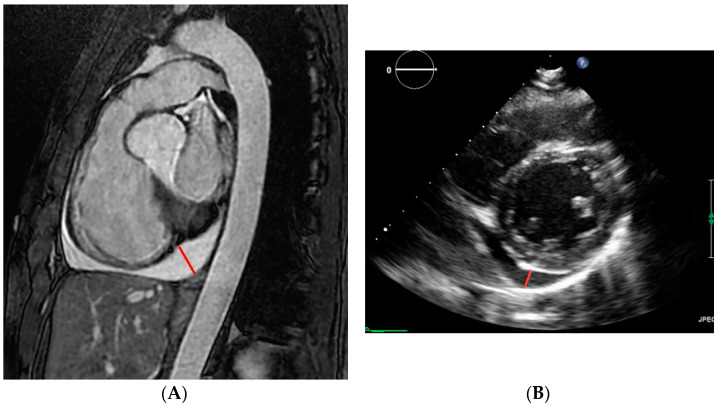
Example of pericardial effusion measurement in ADPKD subjects on (**A**) MRI and (**B**) echocardiography. The measurement of pericardial effusion thickness at end diastole is indicated by the red lines.

Sagittal oblique ECG-gated SSFP MRI images were analyzed by 3 independent, experienced observers (JL, KF, MRP) blinded to all subject information (Figure 2A). Pericardial effusions were quantified by measuring the largest dimension of fluid signal in the pericardial space visible during end diastole. One observer (JL) measured subjects three times in order to assess intraobserver variation. For subjects for whom echocardiography data was available, the thickness of pericardial fluid was measured where it appeared largest during end diastole, as shown in Figure 2B.

### 2.5. Statistical Analysis

All statistical tests were 2-tailed, and a *p*-value < 0.05 was considered statistically significant. Continuous variables were assessed for normality using the Shapiro–Wilk test and summarized as either mean ± standard deviation (SD) for normally distributed variables or median (interquartile range) for variables without normal distribution. Frequency and percentage were calculated for categorical variables.

For two-group matched continuous, normally distributed variables, *t*-test was used. The Mann–Whitney test was used for variables that were not normally distributed to assess the statistical significance at *p*-value < 0.05. For categorical variables, chi-squared test was used to assess statistical significance. The interobserver and intraobserver agreement for measuring pericardial effusion size on MRI was assessed by interclass correlation coefficient (ICC).

Bivariate analysis was used to predict the correlation between the pericardial effusion thickness and the continuous parameters including age, body mass index (BMI), systolic blood pressure, liver volume, kidney volume, spleen volume, cisterna chyli diameter, AST, ALT, albumin, BUN, TSH and eGFR. A multivariate linear regression model was used to predict the mixed effect of the variables found to be significant at *p* < 0.05 level upon bivariate analysis while also including age and gender (male = 0, female = 1).

## 3. Results

### 3.1. Study Population and Characteristics

ADPKD and matched (age, gender, eGFR) control subjects (*n* = 117) were analyzed in this study (Figure 1, Table 2). Data on race were available for matching in 86 of 117 (74%) ADPKD subjects. Demographic and laboratory data of this study are shown in Table 2. Although there was a significant difference in serum albumin between ADPKD and control groups, this difference was minor, and all values were within normal ranges. For the control population, the primary indications for cardiac MRI are listed in Supplemental Table S1. The medications used to control blood pressure in control and ADPKD subjects are listed in Supplemental Table S2. The causes of renal insufficiency in the 44 control subjects with eGFR < 60 mL/min/1.73 m^2^ are listed in Supplemental Table S3. Echocardiography within 1 year of MRI was available in 46 (39%) of the 117 ADPKD subjects.

### 3.2. Interobserver and Intraobserver Variabilities

There was excellent agreement among the three observers for measurement of pericardial effusion on MRI (ICC = 0.939). Intraobserver variability for measuring pericardial effusion on MRI was also excellent (ICC = 0.997).

### 3.3. Prevalence of Pericardial Effusion

Pericardial effusion thickness was larger in ADPKD subjects compared to their matched controls for 59 pairs, smaller for 28 pairs and the same for 30 pairs (Mann–Whitney *p* = 0.001). Pericardial effusion > 5 mm was more prevalent in ADPKD subjects compared to controls (24 of 117 (21%) vs. 4 of 117 (3%), *p* < 0.001) (Table 2). Pericardial effusion > 10 mm showed a strong trend toward greater prevalence in ADPKD subjects compared to controls (5 of 117 (4%) vs. 0 of 117 controls (0%), *p* = 0.06). Comparisons of clinical, laboratory and genotype parameters between ADPKD patients with pericardial effusion ≤5 mm versus >5 mm are shown in Table 3.

Although there were four ADPKD subjects with rheumatologic disease, none of these subjects had pericardial effusion thickness >5 mm. Furthermore, none of the ADPKD subjects with pericardial effusion thickness >5 mm had clinical evidence of heart failure. Increased fluid intake in ADPKD subjects did not correlate with pericardial fluid thickness, as shown in Table 3 and Table 4. One ADPKD subject had mildly elevated TSH, 6.6 mIU/mL, however this patient had only minimal pericardial fluid with a thickness of 1 mm.

### 3.4. Correlation with Laboratory and Imaging Parameters

Upon bivariate linear regression analysis, Table 4, pericardial effusion thickness on MRI of ADPKD subjects significantly correlated with gender, right and left pleural effusion, and negatively correlated with age. There was no correlation with BMI, blood pressure, liver volume, kidney volume, spleen volume, cisterna chyli diameter, AST, ALT, albumin, BUN and eGFR. Multivariate regression analysis (Table 5) including age, gender, and right pleural effusion, showed that female gender (*p* = 0.03) and right pleural effusion (*p =* 0.0000003) were associated with the presence of pericardial effusion (*p* < 0.0001).

### 3.5. Comparing MRI and Echocardiography Measures of Pericardial Effusion

**Figure 3:**
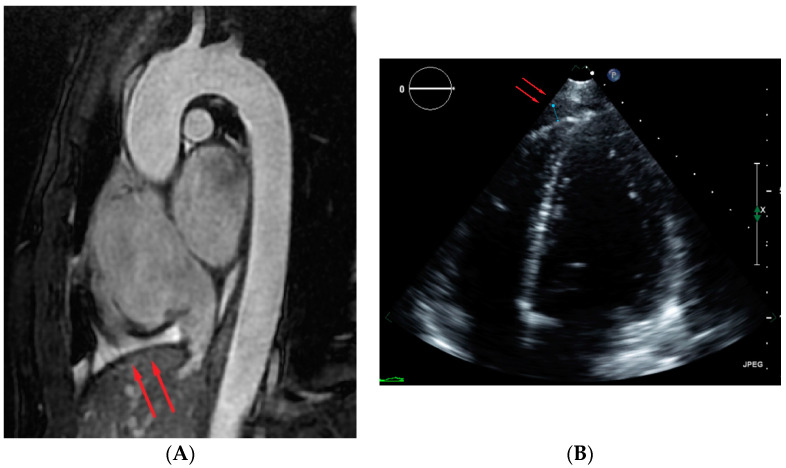
Asymmetrically distributed pericardial effusion (red arrows) on (**A**) MRI and (**B**) echocardiography.

For the 46 ADPKD subjects in whom protocol echocardiography was also available within 1 year of the MRI, on average, the retrospective measurement of pericardial effusion thickness was similar in MRI and echocardiography. In these 46 subjects, there were eight with pericardial effusions >5 mm on MRI. All eight had normal left ventricular ejection fraction. For the three ADPKD subject in whom echo showed a left ventricular ejection fraction <55%, pericardial effusion thickness measured 0, 1 and 1 mm. Although pericardial fluid was found retrospectively on the echocardiography matching the MRI findings in seven subjects, pericardial fluid was prospectively reported in only two of those seven cases. The echocardiography report described a small pericardial effusion in a patient with 6 mm pericardial effusion on MRI and echocardiography described trace pericardial fluid in a patient with a 12 mm pericardial effusion on MRI. In the other five of the seven subjects, echocardiography reported “no pericardial effusion”, although MRI showed pericardial effusions measuring 6, 6, 7, 7 and 8 mm. However, on retrospective review of the echocardiography, pericardial effusions were identified, but asymmetrically distributed more around the right ventricle rather than circumferentially (Figure 3). A summary of other echo findings are provided in Supplemental Table S4; we found no evidence of any difference between ADPKD and control groups in measures of cardiac structure or function.

### 3.6. Genotype

Among 75 ADPKD subjects with genotype data available, 56 were found to have pathogenic mutation of either PKD1 or PKD2—there were 40 subjects with PKD1-only mutation, 14 subjects with PKD2-only mutation and 2 subjects with both PKD1 and PKD2 mutations. Nineteen subjects did not have a pathogenic PKD1 or PKD2 mutation identified despite a clinical diagnosis of ADPKD based upon Pei criteria. Furthermore, 24 of the 40 (60%) PKD1 mutations were truncating and all of the PDK2 mutations were truncating. No statistically significant correlation between genotype and pericardial effusion was detected. Among two subjects who had both PKD1 and PKD2 mutations, one had pericardial effusion > 5 mm.

### 3.7. Clinical Effects of Pericardial Effusion

No subject had a hemodynamically significant pericardial effusion or symptoms attributed to their pericardial effusion. Moreover, no subject required pericardiocentesis or other procedure related to the pericardial effusion.

## 4. Discussion

This retrospective cross-sectional study analyzing ECG-gated cine MRI on 117 ADPKD subjects comparing to age, gender, eGFR matched controls demonstrates that pericardial effusion occurs commonly in ADPKD with a prevalence of 21% compared to 3% in the control population. Multivariate analysis found that female gender and concurrent pleural effusion are associated with the presence of pericardial effusion in ADPKD. Pericardial effusion was observed in subjects with PKD1 and PKD2 mutations.

When comparing echocardiography to ECG-gated MRI, most (seven of eight) pericardial effusions > 5 mm on MRI were retrospectively detected on echocardiography when the reader was aware of MRI results. However, only two of eight effusions seen on MRI were reported on the echocardiography initial reading performed blinded to MRI results. This echocardiography oversight may be due to the location of the effusions around the right ventricle, which is readily evaluated by MRI but not as easily evaluated by echocardiography due to the sternum and ribs limiting acoustic windows. Moreover, pericardial effusion has been only rarely reported in ADPKD subjects [13,14,15]. Qian et al. reported pericardial effusion in ADPKD on CT scans [14]. However, CT is less sensitive and less convincing than MRI for detection of effusions [16,17]. Furthermore, ECG-gated cardiac CT is rarely performed on subjects with ADPKD.

Multivariate analysis found pericardial effusion to be associated with female gender and pleural effusion. The association of pleural and pericardial effusions suggests the possibilities of salt/water overload, heart failure with preserved ejection fraction, serositis or lymphatic problems. Our limited data on fluid intake by ADPKD subjects was not correlated with pericardial effusion thickness. We found no evidence of heart failure or rheumatologic disease in the ADPKD subjects with pericardial effusions > 5 mm. However, the previous finding of an enlarged cisterna chyli in ADPKD subjects [18] raises the possibility of high fluid throughput occurring in the thorax in ADPKD, possibly reflecting increased flow through the lymphatic circulation. Comparison to cisterna chyli diameter in the control subjects was not possible in this study because the cardiac MRI did not include the cisterna chyla within the field of view of T2 weighted images. The absence of a significant association with standard ADPKD imaging biomarkers, including kidney and liver volumes [19,20], suggests pericardial effusion may be an independent ADPKD biomarker corresponding to a different aspect of the disease compared to the other standard imaging and laboratory ADPKD biomarkers that are more focused on other organs, e.g., kidney and liver.

The clinical significance of pericardial effusion in ADPKD patients is not clear. No subject in this study had hemodynamically significant pericardial effusion and none required pericardiocentesis or other therapeutic procedures. However, hemodynamically significant pericardial effusion in an ADPKD patient has been reported [15]. Therefore, follow-up pericardial imaging to assess the progression of an effusion would be reasonable to determine stability. In addition, evaluation of other potential etiologies of a pericardial effusion, (e.g., hypothyroidism, connective tissue diseases), should be considered where clinically indicated. Since the etiology and prognostic value of pericardial effusion are unknown in ADPKD, future research is warranted.

The strengths of this study include a relatively large sample size for this condition, the non-ADPKD control group and prospective analysis of MRI and other demographic/laboratory data from a well-characterized ADPKD population within the Rogosin ADPKD Repository. Limitations of this study include the small number of subjects who underwent both echocardiography and MRI as well as the time interval between those studies, thereby obscuring a comparison of these diagnostic tests for assessing pericardial effusion. The number of subjects evaluated by PKD gene testing was also relatively small, thus, conclusions regarding the possible association of PKD mutation [21] locus on the prevalence of pericardial effusion were not feasible. Further investigation of the relationship of pericardial effusion to genotype in a larger ADPKD cohort is needed. Retrospective analysis of existing research cohort data may have created observer biases and there may have been incomplete medical information for ADPKD and control subjects. Tolvaptan is a type 2 vasopressin receptor antagonist that is approved for the treatment of ADPKD. This aquaretic drug can cause significant dehydration, and consequently, may have attenuated pericardial effusion thickness underestimating the prevalence of pericardial effusion in those ADPKD subjects on tolvaptan.

## 5. Conclusions

This retrospective cross-sectional study showed a relatively high prevalence of pericardial effusion in ADPKD as well as the association of pericardial effusion with female gender and the presence of pleural effusion in ADPKD. The etiology and clinical significance of pericardial effusion in ADPKD is unknown but its association with pleural effusion may have implication regarding the pathophysiology of ADPKD which warrants further investigation.

## Figures and Tables

**Table 1:** MRI imaging parameters.

	Coronal SSFSE ^1^	Axial SSFSE	Axial 3D LAVA ^2^	Axial DWI ^3^	Sagittal SSFP ^4^ Aorta and Heart
Field of view	40–48	30–42	30–42	30–42	30–42
Matrix	320 × 256	320 × 208	288 × 192	160 × 108	240 × 153
Slice thickness	5 mm	5 mm	3 mm	5 mm	7 mm
TR ^5^/TE ^6^/flip	1200/91/130	1000/95/130	3.98/1.24/9	7500/90/90	58/1.2/39

^1^ SSFSE, single-shot fast-spin echocardiography; ^2^ LAVA, liver imaging with volume acceleration; ^3^ DWI, diffusion-weighted imaging; ^4^ SSFP, steady-state free precession; ^5^ TR, repetition time; ^6^ TE, echocardiography time.

**Table 2:** Demographic data in ADPKD subjects with matched controls ^1^.

Characteristic	ADPKD Subjects *n* = 117	Matched Controls *n* = 117	*p* Value
Age	47.3 ± 13	47.8 ± 13	0.74
Male: female	50: 67	50: 67	1
White: Black: Asian: mixed: Native American: unknown	76: 10: 8: 6: 1: 16	85: 11: 5: 6: 1: 9	0.55
Body Mass Index (kg/m^2^)	25 (23–29)	26 (23–30)	0.62
Systolic Blood Pressure (mmHg)	127 (120–136)	125 (112–137)	0.16
Diuretic use	24 (21%)	26 (22%)	0.75
Rheumatological disease	4 (3%)	8 (7%)	0.24
Pericardial effusion (mm) mean ± SD	2.9 ± 3.3	1.6 ± 1.5	0.001
Pericardial effusion > 5 mm	24 (21%)	4 (3%)	0.00006
Estimated Glomerular Filtration Rate (mL/min/1.73 m^2^)	67 (46–93)	69 (52–94)	0.44
Blood Urea Nitrogen (mg/dL)	19 (15–26)	20 (15–27)	0.99
Albumin (g/dL)	4.1 (4–4.3)	4 (3.7–4.3)	0.004
Aspartate Transaminase (U/L)	22 (18–26)	23 (18–31)	0.66
Alanine Transaminase (U/L)	21 (16–28)	25 (16–36)	0.59
Right pleural effusion (mm)	1.8 (0–3.2)	0 (0–0)	<0.001
Left pleural effusion (mm)	0.6 (0–2.8)	0 (0–0.67)	<0.001

^1^ normally distributed parameters and pericardial effusion reported as mean ± standard deviation; non-normally distributed parameters reported as median (interquartile range); categorical parameters reported as number (%).

**Table 3:** Demographic data in ADPKD subjects (pericardial effusion ≤5 mm vs. pericardial effusion >5 mm).

Pericardial Effusion: Characteristic	≤5 mm *n* = 93	>5 mm *n* = 24	*p* Value
Age	49 ± 13	42 ± 11	0.01
Male: female	43: 50	7: 17	0.32
White:Black:Asian:mixed:Native American:unknown	56:7:5:4:1:13	14:2:0:2:0:3	-
Body Mass Index (kg/m^2^)	25 (23–28)	25 (23–30)	0.69
Systolic Blood Pressure (mmHg)	129 ± 14	125 ± 25	0.32
Estimated Glomerular Filtration Rate (mL/min/1.73 m^2^)	65 (45–89)	68 (46–93)	0.72
Right pleural effusion (mm)	3.3 (2.1–4.6)	0.7 (0–2.5)	<0.001
Left pleural effusion (mm)	2.4 (0.4–4)	0 (0–2.6)	0.004
Tolvaptan use	14 (15%)	4 (17%)	0.85
Increased fluid intake (>3 L/day)	35 (38%)	8 (33%)	0.70
Thyroid stimulating hormone (mIU/mL)	1.7 (1.2–2.3)	1.6 (1.1–2.3)	0.58
Blood Urea Nitrogen (mg/dL)	21 (17–30)	19 (15–25)	0.26
Albumin (g/dL)	4.2 (4.1–4.4)	4.1 (3.9–4.3)	0.18
Aspartate Transaminase (U/L)	21 (19–26)	22 (18–26)	0.43
Alanine Transaminase (U/L)	19 (16–30)	21 (15–28)	0.78
Total Kidney Volume/height	994 (660–1305)	803 (519–1470)	0.41
Liver volume (mL)	1895 (1523–2321)	1792 (1517–2125)	0.79
Spleen volume (mL)	246 (208–309)	234 (187–285)	0.24
Cisterna chyli diameter (mm)	4 (3–5)	4 (3–5)	0.84
Genotype data available (%)	59 (63%)	16 (67%)	0.09
PKD1 ^1^-only Mutation (%)	29 of 59 (49%)	11 of 16 (69%)	0.16
Truncating ^2^	20 of 29 (69%)	4 of 11 (36%)	0.07
Nontruncating ^2^	6 of 29 (21%)	5 of 11 (45%)	0.07
PKD2 ^3^-only Mutation (%)	13 of 59 (22%)	1 of 16 (6%)	0.15
Truncating	13 of 13 (100%)	1 of 1 (100%)	1
Nontruncating	0 of 13	0 of 1	1
PKD1 & PKD2 Mutation (%)	1 of 59 (2%)	1 of 16 (6%)	0.32
No mutation detected (%)	16 of 59 (27%)	3 of 16 (19%)	0.49

^1^ PKD1, polycystic kidney disease 1; ^2^ data on truncation was not available for 5 subjects with PKD1 genotyped at other institutions, ^3^ PKD2, polycystic kidney disease 2.

**Table 4:** Bivariate correlations between pericardial effusion thickness and clinical/laboratory variables in ADPKD subjects.

Variables	Correlation Coefficient	*p* Value
Age	−0.28	0.003
Gender	0.24	0.010
Body Mass Index	0.01	0.892
Systolic Blood Pressure	−0.15	0.118
Right pleural effusion	0.50	0.00000001
Left pleural effusion	0.37	0.00004
Total Kidney Volume/height	0.06	0.498
Liver volume	0.06	0.557
Spleen volume	0.13	0.164
Cisterna chyli diameter	0.03	0.787
Serum Creatinine	0.07	0.460
Blood Urea Nitrogen	0.03	0.748
Estimated Glomerular Filtration Rate	−0.04	0.651
Albumin	−0.003	0.970
Aspartate Transaminase	−0.17	0.066
Alanine Transaminase	−0.06	0.497
Thyroid stimulating hormone	−0.06	0.54
Tolvaptan use	−0.03	0.75
Increased fluid intake	−0.14	0.12

**Table 5:** Multivariate analysis including variables with *p* value < 0.05 upon bivariate analysis on ADPKD subjects.

	Coefficient	Standard Error	t Stat	*p*-Value	Lower 95%	Upper 95%
Intercept	2.24	1.25	1.79	0.08	−0.23	4.72
Pleural effusion	0.60	0.11	5.47	0.0000003	0.38	0.81
Age	−0.03	0.02	−1.17	0.24	−0.07	0.02
Gender	1.21	0.54	2.24	0.03	0.14	2.28

## Data Availability

The data presented in this study are available on request from the corresponding author subject to IRB approval. The data are not publicly available due to HIPPA regulations.

## Supplementary Materials

The following supporting information can be downloaded at: https://www.mdpi.com/article/10.3390/jcm11041127/s1, Table S1: Primary indications for Cardiac MRI in control population; Table S2: Medications to control blood pressure in ADPKD and control subjects (note that some subjects are on no blood pressure medications and some are on more than 1 medication so it does not add up to 117 in each column); Table S3: Causes of chronic kidney disease in 44 control subjects with eGFR < 60 mL/min/1.73 m²; Table S4: Cardiac findings in ADPKD subjects with echo within 1 year of MRI and corresponding controls, mean ± standard deviation (min–max).
