# Supplementary material for: Pericardial Effusion on MRI in Autosomal Dominant Polycystic Kidney Disease
Source: J Clin Med. 2022 Feb 21;11(4):1127. doi: 10.3390/jcm11041127 (PMC8879333; doi:10.3390/jcm11041127)
Supplement: Supplementary file 1 [file jcm-11-01127-s001.zip › jcm-1527164-supplementary.pdf]

Supplemental Tables

Supplemental Table S1. Primary indications for Cardiac MRI in control population.

**Primary Cardiac MRI Indication**	**Number of Control Subjects**
Possible cardiomyopathy	59
Arrythmia	11
Hypertension	8
Iron overload	7
Aortic valve disease	5
Possible myxoma or other mass	5
Coronary artery disease	4
Mitral valve disease	3
Stroke	3
Pulmonary valve disease	2
Possible myocarditis	2
Dilated right ventricle	2
Dyspnea	2
Possible pericarditis	1
Atrial septal defect	1
Ventricular septal defect	1
Amyloidosis	1

Supplemental Table S2. Medications to control blood pressure in ADPKD and control subjects (note that some subjects are on no blood pressure medications and some are on more than 1 medication so it does not add up to 117 in each column).

**Blood Pressure Medications**	**Number of ADPKD subjects**	**Number of control subjects**
Beta blockers	19	26
Diuretics	24	26
Calcium channel blockers	23	21
Angiotensin Converting Enzyme Inhibitors	29	14
Angiotensin receptor blockers	44	13
Combined alpha and beta-blockers	0	14
Vasodilators	0	3
Central agonists	0	1

Supplemental Table S3. Causes of chronic kidney disease in 44 control subjects with eGFR<60 *mL/min/1.73 m²*.

**Causes of chronic kidney disease**	**Number of control subjects (eGFR<60** mL/min/1.73 m^2**)**
Diabetes	9
Hypertension without Diabetes	12
Immune mediated	7
Cardiac disease	3
Multiple myeloma in remission	5
dehydration	2
unknown	6

Supplemental Table S4. Cardiac findings in ADPKD subjects with echo within 1 year of MRI and correspoding controls, mean ± standard deviation (min – max).

Parameter	ADPKD	Controls	P value
LV ejection fraction	64 ± 6 (47-78)	63 ± 8 (52-83)	0.27
RV Dimension (cm)	3.6 ± 0.5 (2.8 – 4.4)	3.9 ± 0.8 (2.7 – 5.5)	0.37
Septal Wall Thickness (cm)	0.9 ± 0.2 (0.6 – 1.2)	1 ± 0.2 (0.6 – 1.5)	0.69
LV Posterior Wall thickness (cm)	0.8 ± 0.1 (0.6 – 1.1)	0.9 ± 0.2 (0.6 – 1.2)	0.11
LA Dimension (cm)	3.5 ± 0.6 (2.4 – 4.8)	3.6 ± 0.6 (2.5-5.2)	0.69
LA Volume Index (ml/m^2)	29 ± 8 (15 – 47)	35 ± 15 (15 – 81)	0.16
E/e′ (medial & lateral averaged)	7.7 ± 2.7 (4.4 – 18)	7.8 ± 2.2 (4.1 – 14)	0.43

LV=left ventricle, RV=right ventricle, LA = left atrium, cm = centimeter
